# Infliximab in the treatment of patients with severe COVID-19 (INFLIXCOVID): protocol for a randomised, controlled, multicentre, open-label phase II clinical study

**DOI:** 10.1186/s13063-022-06566-5

**Published:** 2022-09-02

**Authors:** Sina M. Coldewey, Charles Neu, Frank Bloos, Philipp Baumbach, Ulrike Schumacher, Michael Bauer, Philipp Reuken, Andreas Stallmach

**Affiliations:** 1 Department of Anaesthesiology and Intensive Care Medicine, Jena University Hospital, Am Klinikum 1, 07747 Jena, Germany; 2 Septomics Research Centre, Jena University Hospital, Jena, Germany; 3 Centre for Sepsis Control & Care (CSCC), Jena University Hospital, Jena, Germany; 4 Centre for Clinical Studies, Jena University Hospital, Jena, Germany; 5 Clinic for Internal Medicine IV, Jena University Hospital, Jena, Germany

**Keywords:** COVID-19, Infectious diseases, Respiratory infections, Molecular diagnostics, Randomised controlled trial

## Abstract

**Background:**

Despite the intense global research endeavour to improve the treatment of patients with COVID-19, the current therapy remains insufficient, resulting in persisting high mortality. Severe cases are characterised by a systemic inflammatory reaction driven by the release of pro-inflammatory cytokines such as IL-6 and tumour-necrosis-factor alpha (TNF-α). TNF-α-blocking therapies have proved beneficial in patients with chronic inflammatory diseases and could therefore pose a new treatment option in COVID-19. Hitherto, no results from randomised controlled trials assessing the effectiveness and safety of infliximab—a monoclonal antibody targeting TNF-α—in the treatment of COVID-19 have been published.

**Methods:**

In this phase-2 clinical trial, patients with COVID-19 and clinical and laboratory signs of hyperinflammation will be randomised to receive either one dose of infliximab (5 mg/kg body weight) in addition to the standard of care or the standard of care alone. The primary endpoint is the difference in 28-day mortality. Further assessments concern the safety of infliximab therapy in COVID-19 and the influence of infliximab on morbidity and the course of the disease. For the supplementary scientific programme, blood and urine samples are collected to assess concomitant molecular changes. The Ethics Committee of the Friedrich Schiller University Jena (2021-2236-AMG-ff) and the Paul-Ehrlich-Institute (4513/01) approved the study.

**Discussion:**

The results of this study could influence the therapy of patients with COVID-19 and affect the course of the disease worldwide, as infliximab is globally available and approved by several international drug agencies.

**Trial registration:**

The trial was registered at clinicaltrials.gov (NCT04922827, 11 June 2021) and at EudraCT (2021-002098-25, 19 May 2021).

## Background

### Introduction

Coronavirus disease 2019 (COVID-19) caused by the severe acute respiratory syndrome coronavirus 2 (SARS-CoV-2) has led to an ongoing global pandemic. Thus far, despite intense global research, no causal therapy has been established for this severe condition. Although many cases are limited to mild symptoms, there are large inter-individual differences in the course of the disease and 10–20% of hospitalised patients will develop pronounced hypoxia requiring intensive care therapy [1–3]. The cause of these differences remains unknown; however, old age, obesity and pulmonary and cardiovascular conditions are known risk factors for severe COVID-19 [1]. Also, the pulmonary damage of severe cases appears to be inflicted by a hyperinflammatory state. This so-called cytokine storm [4, 5] is characterised by a systemic release of proinflammatory mediators, including, among others, interleukin-6 (IL-6) and tumour-necrosis-factor alpha (TNF-α).

TNF-α is a proinflammatory cytokine produced predominantly by macrophages and regulates a number of inflammatory functions including the release of other cytokines and the induction of fever. In patients with severe COVID-19, the production of TNF-α and IL-6 is sustained by circulating monocytes [6]. It has been shown that the plasma levels of TNF-α correlate with disease severity [7]. Apart from its role in COVID-19, TNF-α plays a major role in several chronic inflammatory diseases and immunosuppressive therapies targeting TNF-α have proved beneficial in these conditions. TNF-α may therefore pose a promising target for the treatment of severe cases of COVID-19 exhibiting hyperinflammation.

Infliximab is a chimeric monoclonal antibody directed against TNF- α. It is able to bind both the soluble and membrane-bound form and induces apoptosis in cells expressing TNF- α, hereby promoting its anti-inflammatory effects (reviewed in [8]). In the European Union, infliximab is approved for rheumatoid arthritis, Crohn’s disease, ulcerative colitis, ankylosing spondylitis, psoriatic arthritis and psoriasis with its clinical efficacy and safety proven in many randomised controlled trials. It has, however, been reported that anti-TNF- α therapy may increase the risk of severe and opportunistic infections such as tuberculosis [9] especially when combined with corticosteroid therapy [10]. Conversely, a meta-analysis showed a lower 28-day mortality following infliximab therapy in sepsis [11].

For COVID-19, the effect of anti-inflammatory and immunomodulatory therapy has been tested with different pharmaceuticals. Part of the standard therapy at the time of publication is the corticosteroid dexamethasone which was introduced following the positive effects on mortality seen in the RECOVERY trial [12]. Tocilizumab, a monoclonal IL-6 antibody, and Janus kinase inhibitors such as baricitinib can be considered in the treatment of patients with moderate disease severity according to the German national guideline for the therapy of COVID-19 [13, 14]. On the other hand, therapy with anakinra, a recombinant IL-1 antagonist is not recommended as it led to increased mortality [15, 16]. In a retrospective analysis of 24 patients treated at Jena University Hospital, we found that patients receiving infliximab showed reduced mortality (14.2% vs. 35.3%) [17]. In a phase-2 proof-of-concept trial, infliximab did not reduce blood C-reactive protein levels in hospitalised patients with COVID-19 when compared with standard therapy [18]. Thus far, however, no published randomised controlled trial analysed the effect of infliximab in addition to standard therapy on mortality, morbidity and the course of the disease in a patient collective with defined hyperinflammation.

### Aim

The overall aim of this randomised, controlled, multicentre, open-label phase II clinical trial is the improvement of the survival of patients with COVID-19 at high risk for severe disease by repurposing a clinically established and globally available anti-inflammatory pharmaceutical for the treatment of a clearly defined subpopulation of patients. Due to the global extent of the pandemic, even small differences in survival or other clinically relevant outcomes could lead to a profound impact on the ability to overcome the pandemic. In addition, the trial integrates a supplementary scientific programme which seeks to collect blood and urine samples to assess concomitant molecular changes in future analyses.

## Methods and analysis

### Study design

**Fig. 1:**
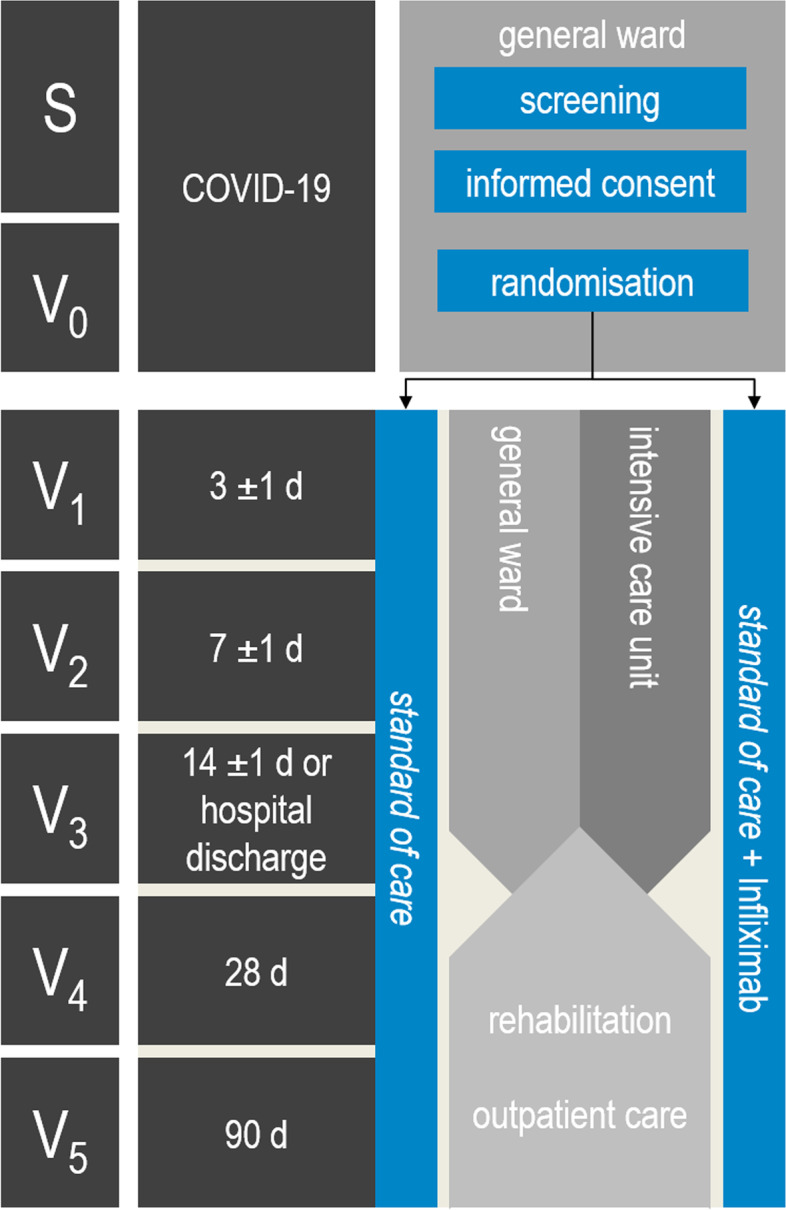
Study design. Hospitalised patients with COVID-19 (positive SARS-CoV-2 PCR testing) with pulmonary symptoms and hyperinflammation are eligible for study inclusion. Patient screening and obtainment of informed consent takes place in the general ward (S). Subsequently, patients are randomly allocated to the control (standard of care, SOC) or interventional group (SOC + infliximab) (*V*_0_). Study events take place 3 ± 1 (*V*_1_), 7 ± 1 (*V*_2_) and 14 ± 1 days after randomisation or at hospital discharge (*V*_3_). Follow-ups take place on days 28 (*V*_4_) and 90 (*V*_5_) after randomisation

This study is a randomised, controlled, multicentre, open-label phase II clinical study. Patient recruitment commenced in July 2021 at Jena University Hospital and aims to enrol 88 patients. The last follow-up is planned for August 2022. Figure 1 summarises the study design including the study events.

### Study objectives and outcome measures

The primary endpoint of this study is the difference in 28-day mortality of patients with COVID-19 receiving one dose of infliximab in addition to the standard of care (interventional group) compared with patients receiving standard of care (control group).

Secondary outcome measures include the assessment of the effect of infliximab on

- The frequency of adverse and severe adverse events by day 90 after randomisation to assess the safety of infliximab in patients with COVID-19
- An excessive inflammatory response
- The morbidity and prognosis of patients with COVID-19
- The incidence of cardiomyopathy on days 3 and 7 after randomisation

as well as the characterisation of the analytical cohort and course of the disease (see also Table 1).

**Table 1:** Secondary endpoints

**Safety of the TNF-α-antibody Infliximab in the treatment of patients with severe COVID-19**	
▶ Frequencies of adverse events (AEs) and serious adverse events (SAEs)	
**Assessment of the effect of infliximab on an excessive immune response in patients with COVID-19**	
▶ Change in the interleukin-6 (IL-6) concentration in blood from randomisation to day 7 (*V*_2_) and day 14 (*V*_3_) after randomisation	
▶ Change in the ferritin concentration in blood from randomisation to day 7 (*V*_2_) and day 14 (*V*_3_) after randomisation	
▶ Change in the lymphocyte count from randomisation to day 7 (*V*_2_) and day 14 (*V*_3_) after randomisation	
**Assessment of the effect of infliximab in patients with severe COVID-19 on morbidity and prognosis**	
▶ Ventilation-free days until day 28 (*V*_4_) after randomisation	
▶ Renal replacement therapy-free days until day 28 (*V*_4_) after randomisation	
▶ Vasopressor-free days until day 28 (*V*_4_) after randomisation	
▶ Rate of occurrence of severe acute respiratory syndrome (ARDS) until day 28 (*V*_4_) after randomisation (Berlin criteria and PaO_2_/FiO_2_ ≤ 100 mmHg with PEEP ≥ 5 cmH_2_O [19])	
▶ WHO-COVID-19-Progression Scale on day 7 (*V*_2_), 14 (*V*_3_) and 28 (*V*_4_) after randomisation	
▶ Rate of admission to the intensive care unit after randomisation up to day 28 (*V*_4_) after randomisation	
▶ Length of hospital stay up to day 28 (*V*_4_) after randomisation	
▶ Length of intensive care unit stay up to day 28 (*V*_4_) after randomisation	
▶Mortality rates at day 14 (*V*_3_) and day 90 (*V*_5_) after randomisation	
▶ EQ5D-3L (health-related quality of life): visual analogue scale value and sub-domain ratings at day 90 (*V*_5_) after randomisation	
▶ EQ5D-3L: index value at day 90 (V_5_) after randomisation	
▶ Frequencies of COVID-19 (long-term) sequelae (positive ratings in checklist)	
▶ Incidence of cardiomyopathy at day 3 (*V*_1_) and/or day 7 (*V*_2_) after randomisation *left-ventricular EF < 52 % in men and < 54 % in women, according to the American Society of Echocardiography* [20] *and the European Association of Cardiovascular Imaging or 10% reduction, if previously reduced* [21–24]	

### Study setting

Patients treated for COVID-19 are recruited in the general ward of up to 10 hospitals in Germany. The list of recruiting centres can be found under ClinicalTrials.gov (NCT04922827).

### Study population

Hospitalised adult patients with COVID-19 (positive SARS-CoV-2 PCR-testing) with typical symptoms of pulmonary involvement and hyperinflammation are eligible for study enrolment, if all inclusion criteria and none of the exclusion criteria are met. Table 2 lists the inclusion and exclusion criteria for study eligibility. The study population consists of two groups, the interventional and the control group.

**Table 2:** Inclusion and exclusion criteria

**Inclusion criteria**	
▶ Age ≥ 18 years	
▶ Infection with SARS-CoV-2 (virus detection by means of a PCR test not older than 72 h)	
▶ Bipulmonary infiltrates (detection by means of chest X-ray or computed tomography)	
▶ COVID inflammation score ≥ 10 [25]	
▶ Serum or plasma ferritin concentration ≥ 500 ng/ml	
▶ Arterial oxygen saturation ≤ 93% when breathing ambient air	
▶ Written informed consent from the patient	
▶ Potentially childbearing women: negative pregnancy test	
**Exclusion criteria**	
***Contraindications for infliximab***	
▶ Allergy to infliximab (or any of the other ingredients of the medication) or to other murine proteins	
▶ Active or latent tuberculosis	
▶ Acute or chronic hepatitis B	
▶ Severe infections such as invasive fungal infections, bacterial sepsis, or abscesses	
▶ Opportunistic infections (e.g. pneumocystosis, listeriosis)	
▶ Moderate or severe heart failure (NYHA class III/IV)	
▶ Immunosuppression (e.g. organ transplantation, AIDS, leukopenia)	
▶ Malignancies or lymphoproliferative diseases or chemotherapy within the last 4 weeks	
▶ Multiple sclerosis or peripheral demyelinating diseases, including Guillain-Barré syndrome	
▶ Treatment with other biologics for approved indications of infliximab (e.g. for rheumatoid arthritis, Crohn’s disease, ulcerative colitis, ankylosing spondylitis, psoriatic arthritis, psoriasis)	
***Further exclusion criteria***	
▶ Autoimmune disease treated with a biological	
▶ Current treatment with TNF-α antibodies, convalescent plasma, bamlanivimab, or other experimental treatments for COVID-19 not considered in national guidelines	
▶ High-flow oxygen therapy, non-invasive/invasive ventilation (WHO-COVID-19 progression scale > 5, [26])	
▶ Pre-existing long-term ventilation or home oxygen therapy	
▶ Child-Pugh C liver cirrhosis	
▶ Pregnancy or breastfeeding	
▶ Life expectancy < 90 days due to other medical conditions	
▶ Limitation or discontinuation of therapy (e.g. refusal of artificial ventilation)	
▶ Participation in another interventional study	
▶ Previous participation in this study	
▶ Interdependence between the patient and the coordinating investigator or other members of the study team	

### Randomisation and intervention

Patients are randomly allocated in a 1:1 ratio to either the control group (standard of care, SOC) or interventional group (SOC + infliximab). Randomisation is performed within 6 hours after study enrolment using an online randomisation tool (PARANDIES) by a member of the study team. Within 3 h after randomisation, infliximab (Remsima®, Celltrion Healthcare, Hungary) is administered intravenously in a dosage of 5 mg/kg body weight over the course of 2 h. The subsequent treatment follows current recommendations for the treatment of COVID-19.

### Study outline

Table 3 summarises all measurements and variables of the study for each scheduled study event based on the SPIRIT guidelines [27]. Baseline and demographic data will be recorded at enrolment (*V*_0_). Recording of clinical data and blood sampling will be performed in the clinical setting at *V*_0_ and 3 ± 1 days (*V*_1_), 7 ± 1 days (*V*_2_) and 14 ± 1 days after randomisation/at hospital discharge (*V*_3_). Transthoracic echocardiography for the assessment of cardiomyopathy will be performed at *V*_1_ and *V*_2_. For ethical reasons, all patients regardless of group allocation will receive the current standard of care for the treatment of COVID-19 at the discretion of the treating physician in accordance with the current national guideline. Patients receiving infliximab should not receive other TNF-α antibodies or experimental treatments. COVID-19-specific therapies will be documented.

**Table 3:** Study outline (SPIRIT)

Domain	sub-domain/variable	S	V_**0**_	V_**1**_	V_**2**_	V_3_	V_**4**_	V_**5**_
**Enrolment**								
screening	inclusion and exclusion criteria, informed consent, pregnancy test	X						
randomisation	randomisation within 6 hours after study enrolment and		X					
**Intervention**								
study medication	start study medication within 3 hours after randomisation		X					
**Assessments**								
adverse events (AEs) and severe AEs			X	X	X	X	X	X
demographic variables	age, sex, body height and weight		X					
(SARS-CoV-2) medical history	date of: first record of SARS-CoV-2 and first symptoms, symptoms of SARS-CoV-2 infection, SARS-Cov-2 immunisation status, date of hospital admission, type of admission, place before patient transfer/admission, nicotine abuse, family history of heart attack, physical activity, accompanying infections		X					
cardiovascular/general comorbidities and previous findings	Charlson comorbidity index, heart attack, congestive heart failure, coronary heart disease, angina pectoris, valvular heart disease, arrhythmias, arterial hypertension, peripheral arterial disease Previous findings echocardiography: rhythm, imaging quality, ejection fraction, pericardial effusion, aortic/mitral/tricuspid/pulmonary valve stenosis and/or insufficiency, VCI diameter, VCI collapse, RAP, SPAP, MPAP, right ventricular function		X					
clinical status and vital signs	COVID-19 inflammation score [25], WHO-COVID-19-Progression Scale [26] consciousness, Glasgow Coma Scale, CAM-ICU, blood pressure, mean arterial blood pressure, heart rate, oxygen saturation and FiO_2_, paO_2_ / FiO_2_ ratio, respiratory rate, urine production		X	X	X	X	X	
organ replacement therapy	administration of oxygen, high-flow oxygen therapy, non-invasive ventilation, invasive ventilation, ECMO, kidney and liver replacement		X	X	X	X		
organ dysfunction	acute encephalopathy, thrombocytopenia, arterial hypoxemia, arterial hypotension, renal dysfunction, metabolic acidosis, septic shock		X	X	X	X		
sepsis-3-criteria	SOFA-score		X	X	X	X		
medication	antiplatelet drugs, anticoagulation, immunosuppressants, angiotensin converting enzyme inhibitors, catecholamines		X	X	X	X		
blood and urine tests: routine	COVID-19-Panel (i.a. troponin, NT-proBNP, PCT, sodium, potassium, chloride, calcium, iron, phosphate, alpha-1antitrypsin, urea, creatinine, bilirubin, albumin, ASAT, ALAT, Gamma-GT, AP, cholinesterase, GLDH, LDH, CK, CK-MB, haptoglobin, haematocrit, haemoglobin, thrombocytes, antithrombin-III, base excess (B.E.) art., bicarbonate (SBC) art., pH art., lactate, Quick, LDL-cholesterol, HDL-cholesterol, HbA1c, IL-6, ferritin, triglycerides, fibrinogen, leucocytes, lymphocytes, D-Dimer, partial thromboplastin time)		X	X	X	X		
blood and urine sampling: supplementary scientific programme	date and time			X	X	X		
virology	SARS-CoV-2 PCR		X	X	X	X		
transthoracic echocardiography (TTE)	rhythm, quality, ejection fraction, pericardial effusion, aortic/mitral/tricuspid/pulmonary valve stenosis and/or insufficiency, VCI diameter, VCI collapse, RAP, SPAP, MPAP, right ventricular function			X	X			
clinical endpoints and cardiovascular events after randomisation	thromboembolic events; cardiovascular events: cardiopulmonary resuscitation, arrhythmia, cardiomyopathy/reduced left ventricular function, STEMI/NSTEMI, angina pectoris, valve stenosis, others			X	X	X	X	X
cumulative endpoints	ventilation-free days, vasopressor-free days, renal replacement therapy-free days, occurrence of severe acute respiratory distress syndrome (Berliner criteria+ PaO_2_/FiO_2_ ≤100 mmHg with PEEP ≥5 cm H_2_O), medication prohibited by the study protocol and SARS-CoV-2-specific therapies						X	
survival status /place of treatment	survival status/date of death, current place of treatment			X	X	X	X	X
general disease progression/patient history after randomisation	current residence, hospital re-admissions, infections, rehabilitation and outpatient therapies						X	X
COVID-19 (long-term) sequelae	checklist (i.a. dyspnoea, taste and smelling disorders, psychological sequela)							X
quality of life: EQ-5D-3L	EQ-5D-3L		X					X
end of study data and cumulative treatment data	irregular end of participation, withdrawal of consent to participate, ICU treatment and length of ICU stay since randomisation, length of hospital stay since hospital admission and since randomisation, pregnancy							X

**V**_**0**_**:** randomisation und administration of Infliximab (interventional group) | **V**_**1**_**:** 3 ± 1 d after randomisation **V**_**2**_**:** 7 ± 1 d after randomisation

**V**_**3**_**:** 14 ± 1 d after randomisation or up to 2 days before planned hospital discharge | **V**_**4**_**:** 28 d (up to 35 d) after randomisation | **V**_**5**_**:** 90 d (up to 97 d) after randomisation

Patients discharged from hospital are monitored by the study team and will be followed up via telephone interviews and questionnaires at 28 and 90 days after randomisation. Aside from checklists for sequelae of COVID-19, health-related quality of life will be assessed via EQ-5D-3L. AEs and SAEs will be assessed and documented at all study events following randomisation.

### Supplementary scientific programme

Within the supplementary scientific programme, the blood and urine samples will be collected and stored for the investigation of translational research questions by analysing biomarkers of organ, metabolic and immunological function and regulation (study visits *V*_1_ through *V*_3_). In addition, the comparison of the course of disease of patients with severe COVID-19 and previously generated datasets from patients with sepsis and healthy subjects is planned [28].

### Sample size and statistical analysis

Sample size planning was based on unpublished retrospective data from Jena University Hospital (April 2020–January 2021, part of the data set in [29]). In 31 patients, the 28-day mortality was 50% in SOC and 12.2% in patients with SOC + infliximab. Under these assumptions, a two-sided Fisher’s exact test at significance level alpha = 0.05 has a power of 95%, if 40 patients are included in each of the two groups. Allowing for a dropout rate of 10%, the total number of patients to be randomised is 88 (2 × 44). We used nQuery 7.0 (Statistical Solutions Ltd, Cork, Ireland) for sample size planning. The full analysis set population (FAS) includes all patients enrolled and randomised to the study with at least one observation after randomisation. Patients will be analysed as randomised (intention-to-treat principle). The primary efficacy analysis will be conducted in the FAS. All variables collected will be analysed in the FAS. The per-protocol collective includes all patients in the intention-to-treat collective who do not have major protocol deviations. In sensitivity analysis, the primary efficacy analysis will be repeated in the per-protocol collective. If there are differences between randomised and actual treatment, an additional ‘as treated’ sensitivity analysis will be performed. An interim-analysis is not planned. Data analysts will be blinded.

### Statistical analysis

In general, all data collected will be analysed using descriptive methods in the two groups and in total. This includes at least the number of collected and missing values, mean, standard deviation, minimum, quartiles including median and maximum for metric variables as well as frequency analyses for ordinal and categorical data. The values of the day of randomisation (*V*_0_) are used as baseline.

The primary endpoint of the study is the mortality rate at day 28 after randomisation. The null hypothesis *H*_0_: π_SOC_=π_SOC+Infliximab_ is tested two-sided against the alternative hypothesis *H*_1_: π_SOC_≠π_SOC+Infliximab_ using a Fisher’s exact test at significance level alpha = 0.05. Absolute and relative frequencies of patients who died are reported for both groups, respectively. The relative risk, the risk difference, and the number needed to treat (NNT) are given as effect measures, each with 95% confidence intervals.

The secondary endpoints will be analysed by appropriate statistical tests depending on the distributional properties of the outcome measures and type of data. Further exploratory analysis may be added, if the results of the descriptive analysis warrant this.

### Dissemination

The Standard Protocol Items: Recommendations for Interventional Trials (SPIRIT) guidelines were followed in the reporting of this study protocol [27]. In order to disseminate the results to the scientific community and health care professionals, the study group will publish and present the results in peer-reviewed journals and at appropriate scientific conferences in accordance with the Consolidated Standards of Reporting Trials (CONSORT) criteria [30]. The reporting of the secondary results and results of the supplementary scientific programme will adhere to the strengthening the reporting of observational studies in epidemiology (STROBE) criteria [31] and transparent reporting of a multivariable prediction model for individual prognosis or diagnosis (TRIPOD) criteria [32], where applicable. Authorship eligibility will follow the recommendations of the International Committee of Medical Journal Editors (ICMJE). For the public and other relevant groups, the results will be disseminated via mass and social media.

The datasets analysed during the current study and statistical code are available from the corresponding author on reasonable request, as is the full protocol.

### Ethics

#### Informed consent

Designated study physicians will obtain written informed consent from patients willing to participate. For the supplementary scientific programme, written informed consent will be obtained separately. Patients incapable of giving informed consent are not eligible for study participation. Patients may withdraw consent at any given time without providing a reason. In this case, some datasets may be continued to be stored and used if necessary either to demonstrate the effect of the medication used, protect legitimate interests of the patient or to comply with the obligation to submit complete drug approval documents in accordance with the German Medicinal Products Act (AMG).

#### Ethics and regulatory approval, study registration and data management

The study in its current version (version 1.0, 10 May 2021) was approved by the Ethics Committee of the Friedrich-Schiller-University Jena (2021-2236-AMG-ff, 27 May 2021) and the Paul-Ehrlich-Institute (4513/01, 27 May 2021) and is in accordance with the Declaration of Helsinki and the AMG. It is registered at ClinicalTrials.gov (NCT04922827) and EudraCT (2021-002098-25). The web-based data entry takes place via an encrypted data link (HTTPS) with pseudonymised patient identification numbers. The data management software (OpenClinica, Waltham, Massachusetts, USA) conforms to the Good Clinical Practice guidelines (21 CFR Part 11). The data is stored on servers of the Centre for Clinical Studies Jena and Jena University Hospital. Patient datasets for collaborating study groups will be transferred in anonymized form. The Centre for Clinical Studies Jena will perform trial monitoring in all study centres according to a monitoring plan. In addition, a data safety monitoring board (DSMB) was established consisting of clinicians and a statistician who are independent from the funding source and study team. The board will convene once per year or when necessary and assist the principal investigator (PI) in evaluating the safety of the study medication according to a DSMB charta. The PI may stop or terminate the study if the recruitment rate is insufficient, new scientific evidence prohibits its continuation or for reasons of patient safety. Any changes to the study protocol must be submitted to and approved by the appropriate committee in an amendment.

## Discussion

This randomised controlled trial includes a targeted approach for the use of an anti-inflammatory therapy with the TNF-α-antibody infliximab in a selected group of patients with COVID-19 and clearly defined hyperinflammation. The multi-centre design facilitates the transferability of study results to hospitals of similar healthcare level. Should infliximab prove to be superior to standard therapy, this could be reflected in a reduced disease severity and mortality. The results of this study could influence the therapy of patients with COVID-19 and affect the course of the disease worldwide, as infliximab is globally available and approved by several international drug agencies.

Due to the novelty of the disease COVID-19 and the transience of the ever-expanding evidence on the effects of new therapeutic approaches on morbidity and mortality, we decided to choose SOC as the comparator to not deny patients state-of-the-art therapeutic options. During the course of this trial, the standard treatment regimens of patients with COVID-19 may change, if new evidence for certain treatments become available. Currently, other anti-inflammatory treatment regimens are being tested in patients with severe cases. Should these become part of the standard of care, this may improve the mortality in patients with clinical deterioration in the control group and hereby affect the primary endpoint of this trial.

The open-label design may overestimate the therapeutic effect due to lack of placebo. However, for ethical reasons, patients in the control SOC-group should receive anti-inflammatory treatments according to the current clinical evidence and recommendations at the discretion of the treating physician in case of clinical deterioration. Here, the open-label study design enables the treating physician to identify which group the patient belongs to without unblinding.

We are aware that the course of the pandemic has been subject to strong fluctuations caused by changing transmissibility and virulence of SARS-CoV-2. This study was planned and initiated at a time when particularly severe cases of COVID-19 resulted in high morbidity and mortality. Currently, we cannot predict whether with increasing population immunity and lower virulence of the prevalent strains the severity of COVID-19 will again increase to such an extent that the recruitment of this study can be completed. However, the planned molecular analyses of the patients’ biomaterials over the course of the disease will result in a gain in scientific knowledge, even if the sample size is not sufficient to answer the primary end-point.

Due to the high incidence of COVID-19 worldwide and the immense effects of the pandemic on societies, health care and economy systems, any progress in the treatment of this new disease would constitute a great success by not only affecting the individual patient but healthcare systems and economies as a whole.

## Trial status

The current version of the study protocol is 1.0 (10 May 2021). Recruitment commenced 18 June 2021. Recruitment is expected to be completed by April 2023.

## Data Availability

No datasets were generated or analysed during the development of the current study protocol. Relevant data will be available upon reasonable request to the corresponding author after publication, while adhering to ethical and data privacy restrictions.

## Abbreviations

AE: Adverse event
AMG: German Medicinal Products Act
ARDS: Acute respiratory disease syndrome
CONSORT: Consolidated Standards of Reporting Trials
COVID-19: Coronavirus disease 2019
DSMB: Data safety monitoring board
FAS: Full analysis set
IL-6: Interleukin-6
PI: Principal investigator
SAE: Severe adverse even
SARS-CoV-2: Severe acute respiratory syndrome coronavirus type 2
STROBE: Strengthening the reporting of observational studies in epidemiology
TNF-α: Tumour necrosis factor alpha
TRIPOD: Transparent reporting of a multivariable prediction model for individual prognosis or diagnosis
